# Plant Enzymes Decrease Prostate Cancer Cell Numbers and Increase TNF-*α In Vivo*: A Possible Role in Immunostimulatory Activity

**DOI:** 10.1155/2019/8103480

**Published:** 2019-07-28

**Authors:** Yeun-Hwa Gu, Takenori Yamashita, Hajime Yamamoto, Tatsuhiko Matsuo, Noriyuki Washino, Jin-Ho Song, Ki-Mun Kang

**Affiliations:** ^1^Department of Radiological Science, Faculty of Health Science, Junshin Gakuen University, Fukuoka, Japan; ^2^Department of Radiological Science, Faculty of Health Science, Suzuka University of Medical Science, Mie, Japan; ^3^Department of Geneinformatics, Graduate School of Pharmaceutical Sciences, Nagoya City University, Nagoya, Japan; ^4^Validux Co., Ltd., 5-1-5 Imaike, Chikusa-Ku, 464-0850 Nagoya, Japan; ^5^Research Institute for Ishokudogen, 9-14-3 Kanbe, Suzuka-City, 513-0801 Mie Pref., Japan; ^6^Department of Radiation Oncology, Seoul St. Mary's Hospital, College of Medicine, The Catholic University of Korea, Seoul, Republic of Korea; ^7^Department of Radiation Oncology, Gyeongsang National University School of Medicine and Gyeongsang National University Changwon Hospital, Changwon, Republic of Korea

## Abstract

Increased caloric intake and Westernized dietary choices may be contributing toward a recent rising trend of incidences of chronic lifestyle-related diseases. In this study, we evaluated the anticancer properties of Plant Enzyme Validux (PEV) using a mouse model. Five-week-old male C3H mice were randomly distributed into four experimental groups: Control, PEV only, 6Gy irradiation only, and PEV + 6Gy. PEV was orally administered daily at 500 mg/kg for 14 days prior to three rounds of 2Gy irradiation. We focused on the anticancer action and immunostimulatory effects of PEV with and without irradiation. Oncogene suppression was observed after PEV treatment as was an increase in TNF-*α*, suggesting an antitumor effect. PEV administration also appeared to reduce oxidative stress as evidenced by a decrease in lipid peroxidation. In addition, PEV confirmed radioprotective effect by radical blocking ability by radiation irradiation. Immunological responses to PEV administration were evidenced by an increase in number of total white blood cells and T lymphocytes. Immunotherapy is drawing more and more attention as a treatment for prostate cancer, suggesting that there will be a need for the identification of specific targets for prostate cancer and for more basic research on the genetic aspects of immunotherapy. Thus, PEV may be of use as a radioprotective supplement during radiotherapy for tumor treatment.

## 1. Introduction

Increasing caloric intake and Westernized dietary choices may be contributing toward a recent rising trend in the incidences of chronic lifestyle-related diseases. Chief among these is cancer, which is one of the leading causes of death [1, 2]. Current clinical regimens in cancer treatment involve surgery, radiation therapy, and chemotherapy, but the accompanying side effects and metastatic recurrence often result in a degradation of patient quality-of-life (QOL) indices [3]. Thus, in recent years, research into immunotherapy using alternative medicines derived from natural substances has made some in-roads due to their less severe side effects [4].

Fermented foods, which have been developed as a means of food preservation, seem to promote health and fitness [4]. The antioxidant and immunomodulatory properties of fermented foods and other natural products have been previously documented [5]. In this context, plant fermentation enzymes have been found to exhibit antitumor effects, such as the upregulation of natural killer (NK) cell activity and increasing the immunopotentiating action of lymphokine activated killer (LAK) cells [5].

In this study, we therefore evaluated the anticancer properties of Plant Enzyme Validux (PEV) using a mouse model. Elevated levels of TNF-*α* were observed in PEV-treated mice 5 weeks after administration, and antitumor activity was also evident since cancer cell proliferation was clearly inhibited by PEV. These results suggest that PEV may exhibit antitumor effects by stimulating TNF-*α* production and NK cell activity [5].

## 2. Materials and Methods

### 2.1. Animals

Five-week-old male BalB/c mice were used for this study, and animals were acclimatized to the facility for one week prior to experimentation. Room temperature was kept at 22 ± 3°C with a humidity of 60%, and water and feed (1 cm2-sized pellet-shaped bait) were provided* ad libitum*.

This study was approved by the Suzuka University of Medical Science Animal Research Ethics Committee (Ref: 18/721/31). Further, the guidelines contained within the Ministry of Education, Culture, Sports, Science and Technology's Notification No. 71 “Basic Guidelines on Implementation of Animal Experiments in Research Organizations” (June 1, 2006) and Ministry of the Environment's “Standards on breeding and storage of experimental animals and relief of pain” were followed.

### 2.2. Experiment Group

Animals were divided into four groups: control (untreated), 2 Gy irradiation, PEV only, and PEV + 2Gy irradiation, with 6-7 animals in each group. Animals were inoculated with SCC-7 cancer cells or in the right femoral region with 4T1 high grade cancer cells from a mouse breast cancer. Daily treatment with PEV began two weeks prior to irradiation. PEV consists of a mixture of vegetable matter, including black soybeans, wheat, rice bran, barley, and rice germs that have been fermented using Aspergillus oryzae and supplemented with seaweed and black sesame as fermented products. This was provided by the* Validux* Corporation.

### 2.3. Irradiation

The source used for irradiation is X-ray radiation. Whole-body radiation exposure (2 Gy) and partial radiation exposure were carried out at a dose of 6 Gy (a dose rate of 1.12 Gy/minute) using an X-ray irradiation device (MG 226 / 4.5, Phillips, Inc., Tokyo, Japan).

### 2.4. Method of Administration

PEV was administered daily by gavage via oral gastrectomy until the end of the experiment. The PEV concentration was 500 mg/kg/day, and the same volume of distilled water was administered to the control groups.

### 2.5. Blood Collection

Blood was collected from the tail vein and into a microtube, and the number of white blood cells and the lymphocytes were measured using an automated hemocytometer. Measurements were performed prior to treatment and 15 days, 16, 19, 21, 28, and 46 days after administration in the nonirradiated groups.

### 2.6. Lipid Peroxidation Measurement

Lipid peroxidation is an important marker of pathophysiological oxidative stress. Since malondialdehyde (MDA) and 4-hydroxynonenal (4-HNE) are formed during lipid peroxidation, they are widely used as indicators of damage from oxidative stress. In this study, MDA was quantified by reaction with thiobarbituric acid (TBA) using a highly sensitive quantification kit to form MDA-TBA complexes. Absorbance (*λ* = 532 nm) or fluorescence (Ex / Em = 532/553 nm) can be used to detect the MDA-TBA complex. Measurement was performed on serum obtained from the fundus of the eye 40 days after irradiation and compared to levels prior to experimentation

### 2.7. Tumor Measurement

The major and minor axes of each tumor were measured every two days from the 4th day after inoculation and tumor volume was calculated by formula (1):

(1) Tumor  volume  mm3=12×major  axis×minor  axis2

### 2.8. Measurement of Tumor Necrosis Factor Alpha (TNF-*α*)

TNF*α* concentration was measured using serum obtained from ocular fundus collection 40 days after irradiation. The experimental group consisted of two groups of enzyme-administered groups orally administered distilled water orally administered enzyme. The administration concentration was 500 mg/kg/day, and each group was 5 animals. The administration period was oral administration for three consecutive weeks.

Measurement of TNF*α* was carried out using a Mouse TNF*α* ELISA kit (Pierce Biotechnology Co., Ltd.) according to the manufacturer's instructions.

### 2.9. Statistics

Experimental results are expressed as mean ± standard deviation. Groups were compared either by ANOVA or Student's* t*-test and data were processed using Stat View software VE 5.0 (HULINKS, Japan) P values of less than 0.05 were deemed to be statistically significant. Experiments were conducted more than once.

## 3. Results 

### 3.1. Effect on Tumor Growth

**Figure 1:**
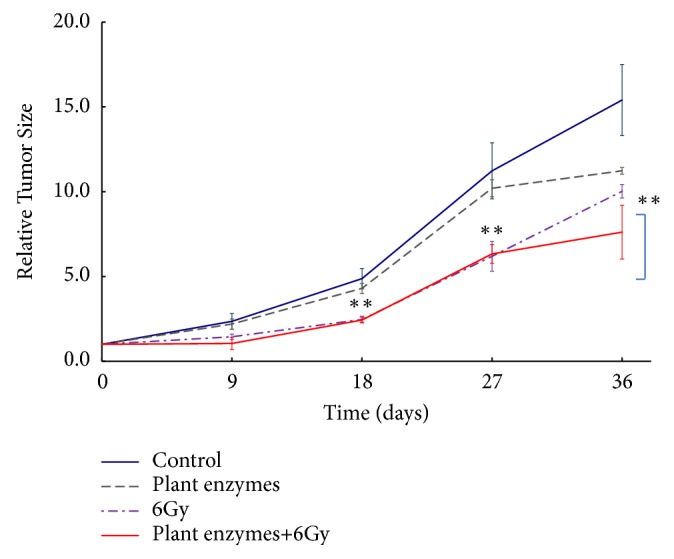
Effect of* Validux Plant Enzyme* on the tumor growth in mice inoculated with 4T1 (high grade) of mouse prostate cancer cell line. Groups of ten mice each were subjected to each treatment. Results represent means ± SD for 7-8 mice. ^*∗∗*^Significantly different from the* Validux Plant Enzyme vs.* control group and* Validux Plant Enzyme* +6Gy* vs.* 6Gy (*P<0.01*).

Tumor volume was calculated using (1) and its variation with time is shown in Figure 1. Substantial tumor growth was observed in the control group, but it was suppressed in the PEV-treated group and was statistically significant after the 18th day following tumor inoculation (*p < 0.01*).

**Figure 2:**
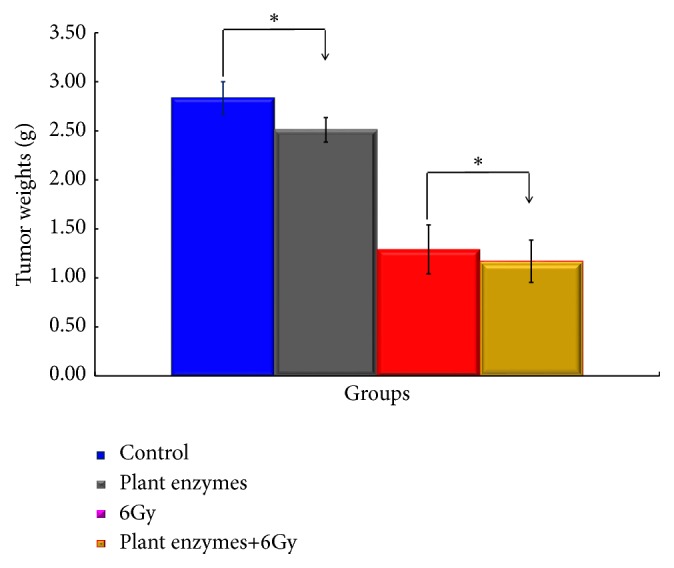
Effect of* Validux Plant Enzyme* on the tumor weight in mice inoculated with 4T1 (high grade) of mouse prostate cancer cell line. Groups of ten mice each were subjected to each treatment. Results represent means ± SD for 7-8 mice. ^*∗*^Significantly different from the* Validux Plant Enzyme vs.* control group and* Validux Plant Enzyme* +6Gy* vs.* 6Gy (*P<0.05*).

The volume of the tumor from the tumor inoculation to the 35th day from irradiation was obtained from (1), and the tumor volume was plotted on the horizontal axis and the number of days elapsed on the vertical axis are shown in Figure 2. Significant differences were found in suppression of tumor growth, tumor volume was small when administered with enzyme, and tumor growth was suppressed (*p < 0.01*).

### 3.2. TNF-*α* Levels

**Figure 3:**
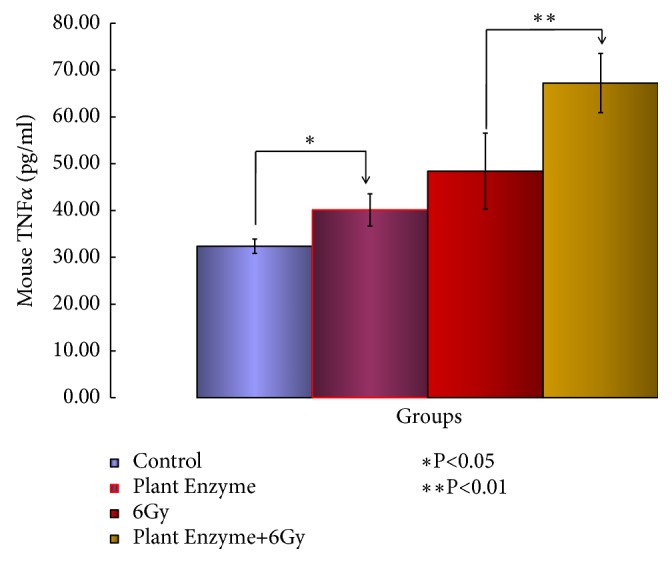
The effects of the* Validux Plant Enzyme* of repeated administration on the levels of TNF-*α* concentration (pg/mouse) in mice with prostate cancer. Changes in the levels were obtained. The results measured represent ± SD for 7-8 mice. TNF-*α* concentration (pg/mouse) was also measured. ^*∗*^Significantly different from the* Validux Plant Enzyme vs.* control group (^*∗*^*P <0.05*) and ^*∗∗*^*Validux Plant Enzyme* +6Gy* vs.* 6Gy (^*∗∗*^*P <0.01*).

There was a marked increase in TNF-*α*in the PEV group with high antitumor effect (Figure 3;* P < 0.01*).

### 3.3. Effect on Blood Lipid Peroxide Levels

**Figure 4:**
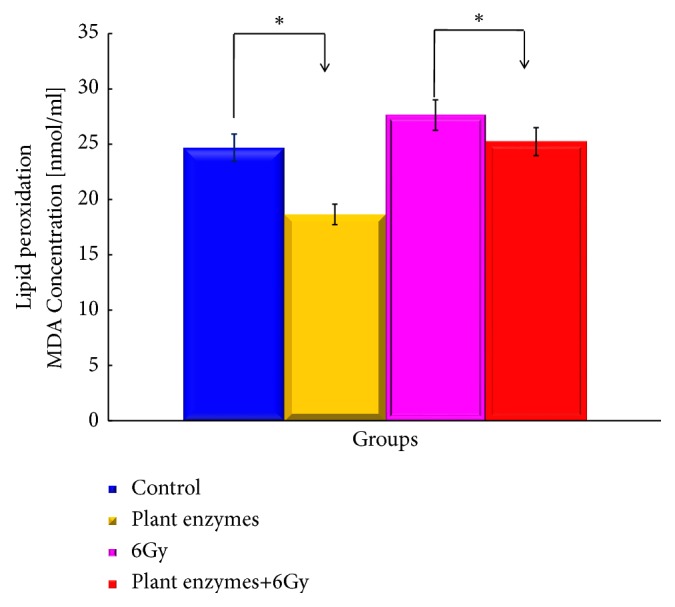
The effects of the* Validux Plant Enzyme* of repeated administration on the levels of lipid peroxidation (MDA concentration) in mice with prostate cancer. Changes in the levels were obtained. The results measured represent ± SD for 7-8 mice. MDA concentration (nmol/ml/mouse) was also measured. ^*∗*^Significantly different from the* Validux Plant Enzyme vs.* control group and* Validux Plant Enzyme* +6Gy* vs.* 6Gy (*P <0.05*).

The effects of the treatments on lipid peroxide (MDA) concentrations in each group are shown in Figure 4.

### 3.4. Influence on Blood Cell Counts

**Figure 5:**
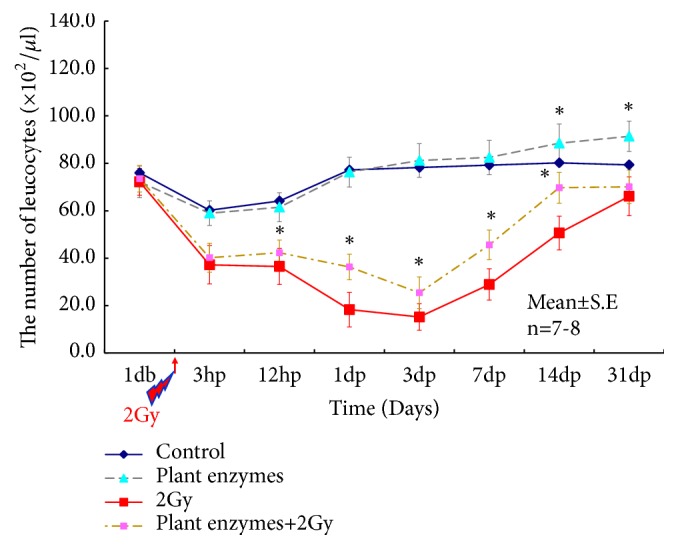
Single-dose effect of* Validux Plant Enzyme *on blood leukocyte counts in mice. There were 7-8 animals in each experimental group. Data are mean ± standard deviation values. Statistically significantly different (^*∗*^*P < 0.05*) from the control group. Statistically significantly different (^*∗*^*P < 0.05*) from the 2Gy group.

**Figure 6:**
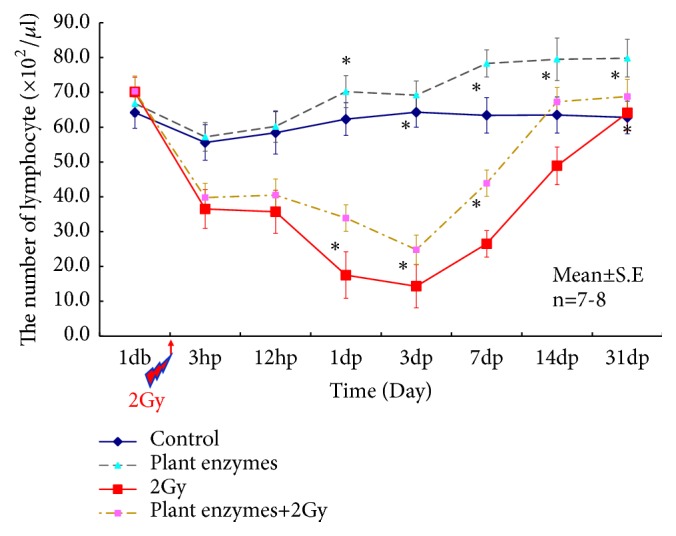
Single-dose effect of* Validux Plant Enzyme *on blood lymphocyte counts in mice. There were 7-8 animals in each experimental group. Data are mean ± standard deviation values. Statistically significantly different (^*∗*^*P < 0.05*) from the control group. Statistically significantly different (^*∗*^*P < 0.05*) from the 2Gy group.

Changes of total leukocyte and lymphocyte counts over time in each group are shown in Figures 5 and 6. There was a significant increase in the overall white blood cell count in the enzyme administration versus the control group (Figure 5;* p<0.05*). The white blood cell count decreased remarkably in the 2 Gy group, but this was suppressed in the PEV + 2 Gy group, where early recovery were observed (*p<0.05*).

There was also a significant increase in the overall lymphocyte count in the PEV group (*p<0.05*) compared to the control group (Figure 6). The number of lymphocytes decreased remarkably in the 2 Gy group, whereas this was suppressed in the PEV + 2 Gy group, where early recovery was observed (*p<0.05*).

## 4. Discussion

### 4.1. Antitumor Effects

Suppression of tumor growth was observed after PEV administration. Lymphocytes are involved in the activation of antitumor activity, and tumors are then suppressed by secondarily activated immune reactions. It is thought that a direct action and a host-mediated action were largely expressed.

It has been suggested that plant enzymes have an immune-activating effect involving cytokines such as IL-2, IL-4 [6], as well was via activation of immunocompetent cells, such as macrophages [7].

Pundir et al. reported that plant enzymes are effective in reducing the risk of cancer [8]. Similarly to the results of this study, plant enzymes are thought to exhibit antitumor effects by mediating the activity of immune cells [8–10].

According to Gu et al., exosomes are extracellular vesicles produced by all cells and exist in various substances. It shows that anticancer action by monocytes and macrophages reinforced by that exosome is present [11].

**Figure 7:**
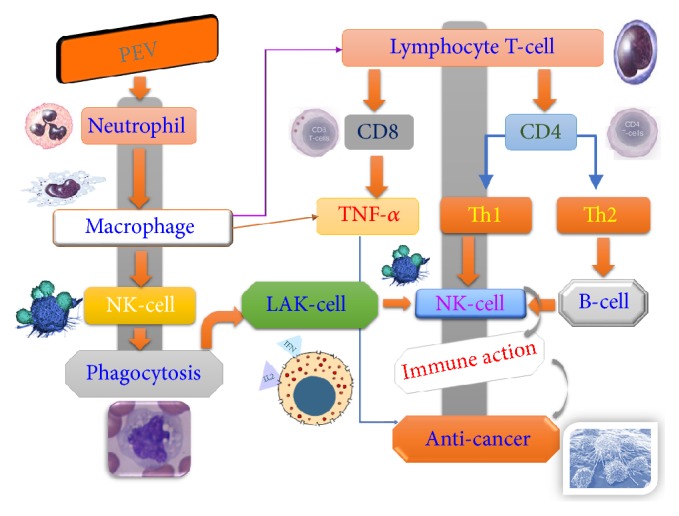
The mechanism of anticancer action of PEV. When PEV is taken into the body from the Peyer's patch, it works strongly on hematopoietic stem cells. Hematopoietic stem cells differentiate into blood cell lineages including granulocytes, monocytes, and NK cells. PEV is an active substance such as lysozyme which is universally present in body fluids. Humoral defense factors such as complement also selectively function in the area of foreign body entry. Next, accumulation of neutrophils from the blood and phagocytosis occurs, and subsequently, accumulation of macrophages is assisted, and phagocytosis of macrophages occurs. Macrophages also activate NK cells and release various cytokines. These things work together to affect the immune response. It is thought that PEV was absorbed into the body and led to antitumor by activation of immune cells that attack malignant tumor cells such as macrophages, NK cells, and TNF-*α*.

Therefore, it is thought that by administration of plant enzymes, from the expression of exosome to immune cell activity, it is related to the antitumor effect. When PEV is taken into the body from the Peyer's patch, it works strongly on hematopoietic stem cells [12]. Hematopoietic stem cells differentiate into blood cell lineages including granulocytes, monocytes, and NK cells [13]. PEV is an active substance such as lysozyme which is universally present in body fluids. Humoral defense factors such as complement also selectively function in the area of foreign body entry. Next, accumulation of neutrophils from the blood and phagocytosis occurs, and subsequently, accumulation of macrophages is assisted, and phagocytosis of macrophages occurs. Macrophages also activate NK cells and release various cytokines [11]. These things work together to affect the immune response. It is thought that PEV was absorbed into the body and led to antitumor by activation of immune cells that attack malignant tumor cells such as macrophages, NK cells, and TNF-*α* [10] (Figure 7).

### 4.2. TNF-*α* Analysis

TNF-*α* is a type of cytokine produced by macrophages that is directly toxic to tumor cells, leading to hemorrhagic tumor necrosis. Its action is extremely potent and effective against the most malignant tumors such as cancer and sarcoma cells while having no effect on normal cells. It also serves as a regulatory factor for controlling immune function, particularly the biological response to infectious diseases and inflammation, and as a switch for promoting the activation of biological response restorative substances and respective body parts [13].

In this study, the production of TNF-*α* in cancer cells was enhanced by PEV, suggesting that TNF-*α* directly caused tumor necrosis and suppressed tumor growth. In addition, the increase in TNF-*α* indicates that macrophages producing TNF-*α* may have been activated [13].

Previously, since malignant tumor cells were thought to be unidentifiable as nonself because their own cells became malignant by mutation, “the immune system does not work for malignant tumors”. It was an established theory. However, continuing research discovered the existence of immune cells, such as NK cells, that attack malignant tumor cells, and the idea that immunity and antitumor effect is involved spread [14, 15].

It is possible therefore that immune activity of PEV observed here was due to an antitumor effect. Therefore, the mechanism of antitumor effect is thought to be on the extension line of the mechanism of immune activity. However, since NK cells were not measured directly, it will be necessary to examine the relationship between the number of NK cells and the antitumor effect in the future.

The increase in TNF-*α* observed in this study strongly supports the fact that PEV has an antitumor effect.

### 4.3. Comparison of Lipid Peroxide Levels

In the study of radioprotective effect on blood cells, it was suggested that suppression of blood cell count reduction accompanying irradiation was due to free radical scavenging action by antioxidants of plant enzymes. However, in order to measure and confirm quantitatively whether or not antioxidant capacity and free radical scavenging activity in peripheral blood is actually increased by administration of plant enzymes, this study can be an indicator of antioxidant activity in peripheral blood in this study Measurement by lipid peroxide (MDA concentration) in serum revealed the effect of lowering lipid peroxide (MDA concentration) in peripheral blood by administration of plant enzymes.

It is thought that enzyme acted as SOD-like activity by the enzyme and eliminated O_2_^−•^. Recently, many results have suggested the involvement of active oxygen as a promoter of skin aging and substances with SOD activity; i.e., that can scavenge active oxygen scavenger. McCord et al. reported that a SOD-like activity in rat liver tissue is measured by NBT (Nitroblue Tetrazolium) reduction and showed an approximately 2 or 3 times SOD-like activity in sample group to which glucan was administered, compared to a nontreated group [16]. Therefore, PEV exhibited SOD-like activity.

### 4.4. Immune Activity of Hemocytes and Radiation Protection

It is known that active radiation and free radicals are generated in the living body due to irradiation, oxidatively damaging lipids, proteins, and nucleic acids, causing various tissue disorders [17, 18]. In addition, in recent years, it has been demonstrated that oxidative damage due to active oxygen and free radicals is intimately involved in various diseases and in aging; its prevention by antioxidants has attracted much attention using both natural and synthetic antioxidants research on plants is being actively conducted in this area [19, 20]. In this study, we examined the antioxidant effect of PEV as well as the immunostimulating, radiation-protection, and the antitumor effects.

Reduction of blood cells is the most problematic side-effect of irradiation. Lymphocytes are highly susceptible to radiation both in mitotic cells in mature cells in peripheral blood. A decrease in lymphocyte count can be detected from as little as a 0.25 Gy. Dose and lymphocytes are the most sensitive cells of mammalian cells and undergo apoptosis only a short time after irradiation [21]. Therefore, in this study, we also examined the radiation-protection effect of PEV and showed that it could suppress the reduction of lymphocytes upon irradiation and also promote their early recovery.

This effect may have been due to antioxidant substances in the PEV. A recent study by Gu et al. showed that plant enzymes reduce oxidative free radicals, thereby alleviating the side effects of radiation therapy [12]. Therefore, it is thought that plant enzymes are effective for reducing side effects of patients during radiotherapy due to the protective effect against infection due to suppression of leucocytopenia and early recovery of hematopoiesis, thus reducing the risk and effective treatment of radiotherapy.

**Figure 8:**
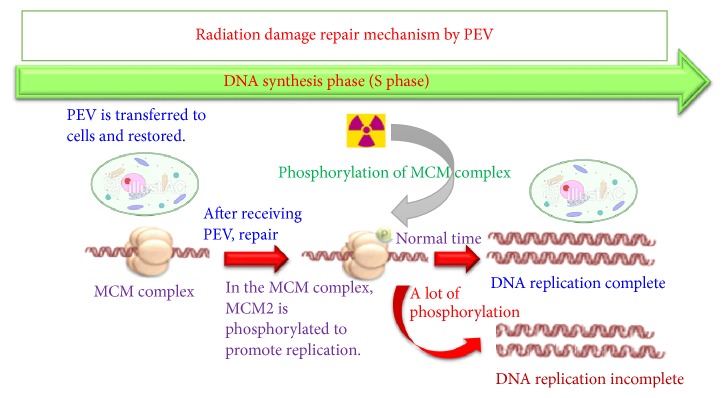
Identify normal DNA replication for radiation damage by PEV and allow progression to the next cleavage in called checkpoint mechanism. DNA that is incompletely replicated by radiation damage is recoverable by PEV and resumed cell division (MCM; mini chromosome maintenance).

Compounds containing SH groups are radical scavengers which have been known to be radioprotective agents for a long time; cysteine and cysteamine are two such compounds [22–24]. Compounds containing S - S bonds, such as cystamine, also have a similar function. However, these protective agents only remove radicals if they are present at the time of irradiation [25, 26]. In addition, due to strong toxicity and side effects, combined side effects due to combination with drugs such as anticancer drugs are regarded as problems [25, 26]. Plant enzymes can be expected to be useful as a radioprotective agent also in terms of promoting repair after a disorder so that it can be seen from the early recovery effect of the number of blood cells after exposure with little side effects (Figure 8).

## 5. Conclusion

In this study, focusing on anticancer action and immunostimulatory effect of PEV, radiation-protection effect and antitumor effect to investigate radiation effects in living organisms were studied. Oncogene length suppression was observed by PEV and an increase in TNF-*α* was observed, confirming the antitumor effect. By the administration of PEV, a decreasing tendency of the organic radical amount was observed due to the action of lowering lipid peroxide (MDA concentration). In addition, PEV confirmed radioprotective effect by radical blocking ability by radiation irradiation. Immunohistochemical activity was observed due to the increase in T cells in leukocyte count and lymphocyte count by PEV administration.

Immunotherapy will be drawing more and more attention for prostate cancer treatment in the future. Therefore, I think that we must search cancer-specific targets for prostate cancer treatment and basic research on immunotherapy from the genetic aspect.

Thus, we would like to propose that PEV may be potentially used as a radioprotective supplement in radiotherapy for tumor suppression.

## Data Availability

The data used to support the findings of this study are available from the corresponding author upon request.
